# Advances in Cuprates and Iron-Based Superconductors: Physics, Properties, and Applications

**DOI:** 10.3390/ma19030483

**Published:** 2026-01-26

**Authors:** Armando Galluzzi, Massimiliano Polichetti

**Affiliations:** Department of Physics “E.R. Caianiello”, University of Salerno, Via Giovanni Paolo II, 132, Fisciano, I-84084 Salerno, Italy; agalluzzi@unisa.it

## Introduction

The study of cuprates and iron-based superconductors continues to evolve rapidly, driven by the quest to understand unconventional pairing mechanisms and to harness their remarkable properties for high-field applications [1,2,3,4,5,6,7,8,9,10]. Despite major progress, key gaps remain in clarifying the microscopic origins of strange metallicity, the role of structural transitions in vortex dynamics, and the optimization of current-carrying capacity in practical conductors. This Special Issue on “Advances in Cuprates and Iron-Based Superconductors: Physics, Properties, and Applications”, addresses these challenges through six contributions that span theory, experimental study, and application. Together, they provide new insights into anomalous transport, vortex behavior, scalable fabrication routes, and interfacial phenomena, bridging fundamental physics with engineering solutions. Looking forward, future research should integrate advanced spectroscopic probes with theoretical modeling; explore heterostructures and interfacial effects to enhance superconducting performance; and develop scalable, cost-effective synthesis methods to fully realize the potential of these materials in next-generation quantum and energy technologies. This Editorial summarizes the six publications (five research articles and one review) included in this Special Issue.

The article on “The Shrinking Fermi Liquid Scenario for Cuprates Under the Scrutiny of Optical Conductivity Measurements” [11] analyzes the strange metallic state of cuprates. While optical conductivity data have often been interpreted through marginal Fermi liquid (MFL) theory [12,13], the authors propose a shrinking Fermi liquid (SFL) scenario [14,15] based on experimentally observed charge density fluctuations (CDFs) [16,17]. Within this framework, increased damping of CDFs at low temperatures shrinks the Fermi liquid regime, extending strange metal behavior down to very low temperatures [18]. Unlike MFL, SFL predicts finite quasiparticle mass and only approximate ω/T scaling, yet still reproduces optical conductivity features when combined with higher-energy excitations such as phonons and paramagnons [19]. The article’s conclusion states that optical experiments cannot uniquely distinguish MFL from SFL, making the latter a compelling, microscopically grounded alternative.

The article on “Magnetic Memory Effects in BaFe_2_(As_0.68_P_0.32_)_2_ Superconducting Single Crystal” [20] explores vortex dynamics in iron-based superconductors. The 122 family is notable for its high T_c_ and large upper critical fields [21,22], with P substitution inducing superconductivity and quantum critical behavior [23,24]. In slightly overdoped BaFe_2_(As_0.68_P_0.32_), besides the second magnetization peak (SMP), an anomalous peak effect in temperature appears, and is linked to the rhomb-to-square transition (RST) of the Bragg vortex glass [25,26]. In this work, multi-harmonic AC susceptibility under different cooling protocols revealed a distinct temperature region with a magnetic memory effect, reflecting the direction of the RST. Large differences between low- and high-frequency third harmonic signals show that the structural transition evolves on a slower timescale than vortex excitations. The paper concludes that RST strongly influences vortex dynamics beyond the SMP onset.

The article on “Iron-Based Superconductors for High-Field Applications: Realization of High Engineering Critical Current Density” [27] reports advances in Ba_0.6_K_0.4_Fe_2_As_2_ (Ba-122) tapes for high-field magnets. In general, iron-based superconductors combine high upper critical fields, low anisotropy, and cost-effective PIT fabrication [28,29,30]. In this work, by optimizing hot pressing pressure and increasing the filling factor to ~40%, silver-sheathed Ba-122 tapes achieved a record engineering critical current density (J_e_) of 4.1 × 10^4^ A/cm^2^ at 4.2 K and 10 T, the highest ever reported for Ba-122 conductors [31,32]. Microstructural analyses (XRD, SEM, and EBSD) showed improved grain size, density, and c-axis texture at optimal pressure, while excessive pressure introduced cracks. The results demonstrate that balancing the filling factor and microstructure is crucial, confirming Ba-122 tapes as strong candidates for practical applications in high-field technologies.

The paper on “Exploring Unconventional Electron Distribution Patterns: Contrasts Between FeSe and FeSe/STO Using an Ab Initio Approach” investigates the puzzling electron distribution observed in the ARPES spectra of iron-based superconductors [33,34]. Using ab initio calculations, the authors analyze the interplay of antiferromagnetism (AFM), spin-density waves (SDWs), charge-density waves (CDWs), and differential phonons [35,36]. In FeSe, these synergistic effects enhance electron–phonon coupling to values comparable with the ARPES energy range (~30–300 meV). In FeSe/SrTiO_3_, stronger interfacial phonons and SDW/CDW effects push the coupling to ~0.5 eV, matching experimental ARPES data [37,38]. The study concludes that magnetoelectric pulses from SDW/CDW phenomena may trigger the unconventional ARPES energy range, offering a microscopic explanation.

The article “Fe(Se,Te) Thin Films Deposited through Pulsed Laser Ablation from Spark Plasma Sintered Targets” presents Fe(Se,Te) thin films grown by pulsed laser deposition (PLD) using spark plasma-sintered (SPS) targets. As already reported, iron-based superconductors are promising for high-field applications due to thei high critical fields and low anisotropy, but the use of SPS enables the rapid densification of Fe(Se,Te) polycrystals, yielding dense, well-connected pellets with T_c,onset_ ≈ 16 K [39,40,41]. The authors prove that films deposited on CaF_2_ substrates show T_c,onset_ ≈ 18 K, high upper critical fields with low anisotropy, and critical current densities above 10^5^ A/cm^2^ and up to 16 T. Pinning analysis reveals point-like pinning centers, confirming the excellent film quality. The work demonstrates SPS to be a scalable route to reproducible Fe(Se,Te) targets for coated conductors [42,43,44].

The review “Properties and Applications of Iron-Chalcogenide Superconductors” summarizes the fabrication, properties, and applications of iron–chalcogenide superconductors. The 11 systems (FeSe, Fe(Se,Te)) offer simple, non-toxic structures and tunable superconductivity [45,46,47]. Techniques such as annealing, intercalation, ammonothermal synthesis, and ionic gating raised T_c_ values up to 48 K [48,49,50], while single-layer FeSe films on SrTiO_3_ reach record T_c_ ≈ 109 K [51]. Critical current density and flux pinning are discussed for single crystals and films, with coated conductors achieving meter-long lengths. Applications include superconducting radio frequency cavities, coils, and high-field magnets. The review highlights iron–chalcogenide as both a theoretical platform and as a practical candidate for next-generation superconducting technologies.

In conclusion, the six contributions collected in this Special Issue highlight both the fundamental advances and practical progress achieved in the study of cuprates and iron-based superconductors. From new theoretical frameworks for strange metallicity to experimental insights into vortex dynamics, and from innovative synthesis routes to record-setting current densities, these works collectively bridge the gap between microscopic understanding and technological application. They demonstrate how interdisciplinary approaches combining spectroscopy, modeling, and scalable fabrication are essential to addressing existing challenges. Looking ahead, future research should focus on integrating fundamental physics with engineering innovation, exploring heterostructures and interfacial phenomena, and developing cost-effective synthesis methods to fully unlock the transformative potential of these materials in quantum technologies and high-field applications.

## References

[1] Kwok W.K., Welp U., Glatz A., Koshelev A.E., Kihlstrom K.J., Crabtree G.W. (2016). Vortices in high-performance high-temperature superconductors. Rep. Prog. Phys..

[2] Blatter G., Feigel’Man M.V., Geshkenbein V.B., Larkin A.I., Vinokur V.M. (1994). Vortices in high-temperature superconductors. Rev. Mod. Phys..

[3] Biju A., Vinod K., Aloysius R.P., Syamaprasad U. (2007). Improved superconducting properties by La addition in (Bi,Pb)-2212 bulk superconductor. J. Alloys Compd..

[4] Belala K., Galluzzi A., Mosbah M.F., Polichetti M. (2021). Transport and magnetic properties of Bi(Pb)2212 superconducting ceramics doped by low rate of potassium. Mater. Sci..

[5] Miura N., Nakagawa H., Sekitani T., Naito M., Sato H., Enomoto Y. (2002). High-magnetic-field study of high-Tc cuprates. Phys. B Condens. Matter.

[6] Hosono H., Yamamoto A., Hiramatsu H., Ma Y. (2018). Recent advances in iron-based superconductors toward applications. Mater. Today.

[7] Polichetti M., Galluzzi A., Kumar R., Goyal A. (2025). Equation for Calculation of Critical Current Density Using the Bean’s Model with Self-Consistent Magnetic Units to Prevent Unit Conversion Errors. Materials.

[8] Fiamozzi Zignani C., Corato V., Leo A., De Marzi G., Mancini A., Takano Y., Yamashita A., Polichetti M., Galluzzi A., Rufoloni A. (2016). Fabrication and Characterization of Sintered Iron-Chalcogenide Superconductors. IEEE Trans. Appl. Supercond..

[9] Galluzzi A., Buchkov K., Tomov V., Nazarova E., Leo A., Grimaldi G., Pace S., Polichetti M. (2020). Mixed state properties analysis in AC magnetic field of strong pinning Fe(Se,Te) single crystal. Supercond. Sci. Technol..

[10] Aswathy P.M., Anooja J.B., Sarun P.M., Syamaprasad U. (2010). An overview on iron based superconductors. Supercond. Sci. Technol..

[11] Caprara S., Di Castro C., Mirarchi G., Seibold G., Grilli M. (2024). The Shrinking Fermi Liquid Scenario for Cuprates Under the Scrutiny of Optical Conductivity Measurements. Materials.

[12] Varma C.M., Littlewood P.B., Schmitt-Rink S., Abrahams E., Ruckenstein A.E. (1989). Phenomenology of the normal state of Cu-O high-temperature superconductors. Phys. Rev. Lett..

[13] Littlewood P.B., Varma C.M. (1991). Phenomenology of the normal and superconducting states of a marginal Fermi liquid. J. Appl. Phys..

[14] Caprara S., Di Castro C., Mirarchi G., Seibold G., Grilli M. (2022). Dissipation-driven strange metal behavior. Commun. Phys..

[15] Grilli M., Di Castro C., Mirarchi G., Seibold G., Caprara S. (2023). Dissipative Quantum Criticality as a Source of Strange Metal Behavior. Symmetry.

[16] Arpaia R., Caprara S., Fumagalli R., De Vecchi G., Peng Y.Y., Andersson E., Betto D., De Luca G.M., Brookes N.B., Lombardi F. (2019). Dynamical charge density fluctuations pervading the phase diagram of a Cu-based high-Tc superconductor. Science.

[17] Arpaia R., Martinelli L., Sala M.M., Caprara S., Nag A., Brookes N.B., Camisa P., Li Q., Gao Q., Zhou X. (2023). Signature of quantum criticality in cuprates by charge density fluctuations. Nat. Commun..

[18] Fournier P., Mohanty P., Maiser E., Darzens S., Venkatesan T., Lobb C.J., Czjzek G., Webb R.A., Greene R.L. (1998). Insulator-metal crossover near optimal doping in Pr_2−x_Ce_x_CuO_4_: Anomalous normal-state low temperature resistivity. Phys. Rev. Lett..

[19] Allen P.B. (2015). Electron self-energy and generalized Drude formula for infrared conductivity of metals. Phys. Rev. B-Condens. Matter Mater. Phys..

[20] Badea A.M., Ivan I., Miclea C.F., Crisan D.N., Galluzzi A., Polichetti M., Crisan A. (2024). Magnetic Memory Effects in BaFe_2_(As_0.68_P_0.32_)_2_ Superconducting Single Crystal. Materials.

[21] Rotter M., Tegel M., Johrendt D. (2008). Superconductivity at 38 K in the iron arsenide (Ba_1−x_K_x_)Fe_2_As_2_. Phys. Rev. Lett..

[22] Altarawneh M.M., Collar K., Mielke C.H., Ni N., Bud’Ko S.L., Canfield P.C. (2008). Determination of anisotropic H_c2_ up to 60 T in Ba_0.55_K_0.45_Fe_2_As_2_ single crystals. Phys. Rev. B-Condens. Matter Mater. Phys..

[23] Demirdiş S., Fasano Y., Kasahara S., Terashima T., Shibauchi T., Matsuda Y., Konczykowski M., Pastoriza H., Van Der Beek C.J. (2013). Disorder, critical currents, and vortex pinning energies in isovalently substituted BaFe_2_(As_1−x_P_x_)_2_. Phys. Rev. B-Condens. Matter Mater. Phys..

[24] Hashimoto K., Cho K., Shibauchi T., Kasahara S., Mizukami Y., Katsumata R., Tsuruhara Y., Terashima T., Ikeda H., Tanatar M.A. (2012). A sharp peak of the zero-temperature penetration depth at optimal composition in BaFe_2_(As_1−x_P_x_)_2_. Science.

[25] Miu L., Ionescu A.M., Miu D., Burdusel M., Badica P., Batalu D., Crisan A. (2020). Second magnetization peak, rhombic-to-square Bragg vortex glass transition, and intersecting magnetic hysteresis curves in overdoped BaFe_2_(As_1−x_P_x_)_2_ single crystals. Sci. Rep..

[26] Galluzzi A., Crisan A., Badea Ionescu A.M., Ivan I., Leo A., Grimaldi G., Polichetti M. (2025). Multiharmonic AC magnetic susceptibility analysis of the rhombic-to-square transition in the Bragg vortex glass phase in a BaFe_2_(As_1−x_P_x_)_2_ crystal. Results Phys..

[27] Yang P., Huang H., Han M., Liu C., Yao C., Ma Y., Wang D. (2024). Iron-Based Superconductors for High-Field Applications: Realization of High Engineering Critical Current Density. Materials.

[28] Galluzzi A., Buchkov K., Nazarova E., Tomov V., Grimaldi G., Leo A., Pace S., Polichetti M. (2019). Transport properties and high upper critical field of a Fe(Se,Te) iron based superconductor. Eur. Phys. J. Spec. Top..

[29] Grimaldi G., Leo A., Martucciello N., Braccini V., Bellingeri E., Ferdeghini C., Galluzzi A., Polichetti M., Nigro A., Villegier J.-C. (2019). Weak or Strong Anisotropy in Fe(Se,Te) Superconducting Thin Films Made of Layered Iron-Based Material?. IEEE Trans. Appl. Supercond..

[30] Togano K., Gao Z., Matsumoto A., Kikuchi A., Kumakura H. (2017). Fabrication of (Ba,K)Fe_2_As_2_ tapes by ex situ PIT process using Ag-Sn alloy single sheath. Supercond. Sci. Technol..

[31] Huang H., Yao C., Dong C., Zhang X., Wang D., Cheng Z., Li J., Awaji S., Wen H., Ma Y. (2018). High transport current superconductivity in powder-in-tube Ba_0.6_K_0.4_Fe_2_As_2_ tapes at 27 T. Supercond. Sci. Technol..

[32] Gao Z., Togano K., Zhang Y., Matsumoto A., Kikuchi A., Kumakura H. (2017). High transport Jc in stainless steel/Ag-Sn double sheathed Ba122 tapes. Supercond. Sci. Technol..

[33] Jia X.W., Liu H.Y., Zhang W.T., Zhao L., Meng J.Q., Liu G.D., Dong X.L., Wu G., Liu R.H., Chen X.H. (2008). Common features in electronic structure of the oxypnictide superconductors from photoemission spectroscopy. Chin. Phys. Lett..

[34] Hagiwara K., Ishikado M., Horio M., Koshiishi K., Nakata S., Ideta S., Tanaka K., Horiba K., Ono K., Kumigashira H. (2021). Superconducting gap and pseudogap in the surface states of the iron-based superconductor PrFeAsO_1−y_ studied by angle-resolved photoemission spectroscopy. Phys. Rev. Res..

[35] Coh S., Cohen M.L., Louie S.G. (2016). Antiferromagnetism enables electron-phonon coupling in iron-based superconductors. Phys. Rev. B.

[36] Coh S., Cohen M.L., Louie S.G. (2015). Large electron-phonon interactions from FeSe phonons in a monolayer. New J. Phys..

[37] Zhang C., Liu Z., Chen Z., Xie Y., He R., Tang S., He J., Li W., Jia T., Rebec S.N. (2017). Ubiquitous strong electron-phonon coupling at the interface of FeSe/SrTiO_3_. Nat. Commun..

[38] Tang C., Liu C., Zhou G., Li F., Ding H., Li Z., Zhang D., Li Z., Song C., Ji S. (2016). Interface-enhanced electron-phonon coupling and high-temperature superconductivity in potassium-coated ultrathin FeSe films on SrTiO_3_. Phys. Rev. B.

[39] Palenzona A., Sala A., Bernini C., Braccini V., Cimberle M.R., Ferdeghini C., Lamura G., Martinelli A., Pallecchi I., Romano G. (2012). A new approach for improving global critical current density in Fe(Se_0.5_Te_0.5_) polycrystalline materials. Supercond. Sci. Technol..

[40] Tokuta S., Hasegawa Y., Shimada Y., Yamamoto A. (2022). Enhanced critical current density in K-doped Ba122 polycrystalline bulk superconductors via fast densification. iScience.

[41] Olevsky E.A., Bradbury W.L., Haines C.D., Martin D.G., Kapoor D. (2012). Fundamental aspects of spark plasma sintering: I. Experimental analysis of scalability. J. Am. Ceram. Soc..

[42] Obradors X., Puig T. (2014). Coated conductors for power applications: Materials challenges. Supercond. Sci. Technol..

[43] Sylva G., Augieri A., Mancini A., Rufoloni A., Vannozzi A., Celentano G., Bellingeri E., Ferdeghini C., Putti M., Braccini V. (2019). Fe(Se,Te) coated conductors deposited on simple rolling-assisted biaxially textured substrate templates. Supercond. Sci. Technol..

[44] Sylva G., Bellingeri E., Bernini C., Celentano G., Ferdeghini C., Leveratto A., Lisitskiy M., Malagoli A., Manca N., Mancini A. (2020). The role of texturing and thickness of oxide buffer layers in the superconducting properties of Fe(Se,Te) Coated Conductors. Supercond. Sci. Technol..

[45] Hsu F.-C., Luo J.-Y., Yeh K.-W., Chen T.-K., Huang T.-W., Wu P.M., Lee Y.-C., Huang Y.-L., Chu Y.-Y., Yan D.-C. (2008). Superconductivity in the PbO-type structure α-FeSe. Proc. Natl. Acad. Sci. USA.

[46] Fang M.H., Pham H.M., Qian B., Liu T.J., Vehstedt E.K., Liu Y., Spinu L., Mao Z.Q. (2008). Superconductivity close to magnetic instability in Fe(Se_1−x_Te_x_)_0.82_. Phys. Rev. B.

[47] Sun Y., Shi Z., Tamegai T. (2019). Review of annealing effects and superconductivity in Fe_1+y_Te_1−x_Se_x_ superconductors. Supercond. Sci. Technol..

[48] Ying T.P., Chen X.L., Wang G., Jin S.F., Zhou T.T., Lai X.F., Zhang H., Wang W.Y. (2012). Observation of superconductivity at 30~46 K in A_x_Fe_2_Se_2_(A = Li, Na, Ba, Sr, Ca, Yb and Eu). Sci. Rep..

[49] Guo J., Lei H., Hayashi F., Hosono H. (2014). Superconductivity and phase instability of NH_3_-free Na-intercalated FeSe_1−z_S_z_. Nat. Commun..

[50] Hatakeda T., Noji T., Sato K., Kawamata T., Kato M., Koike Y. (2016). New alkali-metal-And 2-phenethylamine-intercalated superconductors A_x_(C_8_H_11_N)_y_Fe_1−z_Se (A = Li, Na) with the largest interlayer spacings and Tc 40K. J. Phys. Soc. Jpn..

[51] Ge J.-F., Liu Z.-L., Liu C., Gao C.-L., Qian D., Xue Q.-K., Liu Y., Jia J.-F. (2014). Superconductivity above 100 K in single-layer FeSe films on doped SrTiO_3_. Nat. Mater..

